# Supplementary material for: Single-cell analysis of human ovarian cortex identifies distinct cell populations but no oogonial stem cells
Source: Nat Commun. 2020 Mar 2;11:1147. doi: 10.1038/s41467-020-14936-3 (PMC7052271; doi:10.1038/s41467-020-14936-3)
Supplement: Supplementary file 1 — Supplementary Information [file 41467_2020_14936_MOESM1_ESM.pdf]

Single-cell analysis of human ovarian cortex identifies distinct cell populations but no oogonial stem cells.

Magdalena Wagner, Masahito Yoshihara, Iyadh Douagi, Anastasios Damdimopoulos, Sarita Panula, Sophie Petropoulos, Haojiang Lu, Karin Pettersson, Kerstin Palm, Shintaro Katayama, Outi Hovatta, Juha Kere, Fredrik Lanner*, Pauliina Damdimopoulou*.

INDEX

SUPPLEMENTARY FIGURES
Supplementary Figure 1 **Page 2**
ScRNA-seq analysis of unsorted ovarian cortical cells.
Supplementary Figure 2 **Page 3**
Sorted ovarian DDX4 Ab+ and Ab- cortex cells.
Supplementary Figure 3 **Page 5**
Validation of markers identified in transcriptomic and proteomic analysis.
Supplementary Figure 4 **Page 7**
Germline-like cells in the adult human ovary.

SUPPLEMENTARY TABLES
Supplementary Table 1
First surface marker screening of human ovarian cortex cells. Page 8
Second surface marker screening of human ovarian cortex cells. Page 13
Third surface marker screening of human ovarian cortex cells. Page 15

SUPPLEMENTARY DATA Page 17
Separately attached as xlsx files.
Supplementary Data 1
Cell Ranger metrics and gene expression analysis of unsorted ovarian cortex cells.
Supplementary Data 2
Cell Ranger metrics and gene expression analysis of sorted ovarian cortex cells.
Supplementary Data 3
Upregulated genes in cultured human ovarian DDX4 Ab+ and DDX4 Ab- cells.

REFERENCES Page 18

Supplementary Figure 1. ScRNA-seq analysis of unsorted ovarian cortical cells.

a

	GRP	C-sec
oocyte	4	14
immune	19	29
granulosa	5	132
endothelial	140	494
perivascular	422	785
stroma	5,125	4,991
total	5,715	6,445

b

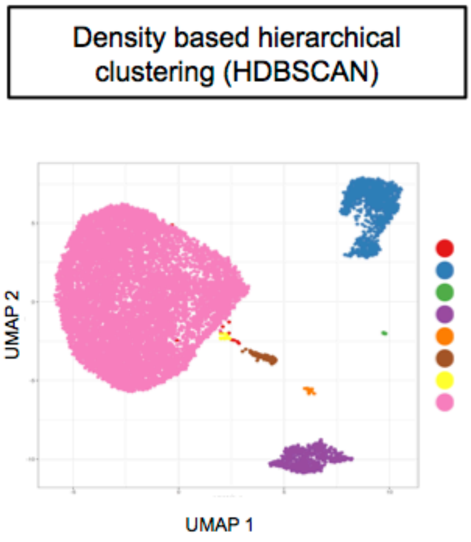

c

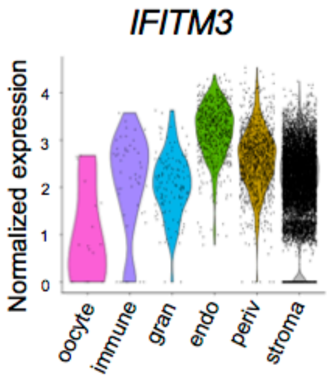

Supplementary Figure 1. ScRNA-seq analysis of unsorted ovarian cortical cells. a, Number of cells contributing to the different cell clusters from GRP and C-sec tissue (in Fig. 2b). **b**, Confirmation of cluster identification using HDBSCAN. **c**, Violin plot showing the expression levels of *IFITM3* in unsorted ovarian cortex cells.

Supplementary Figure 2. Sorted ovarian DDX4 Ab+ and Ab- cortex cells.

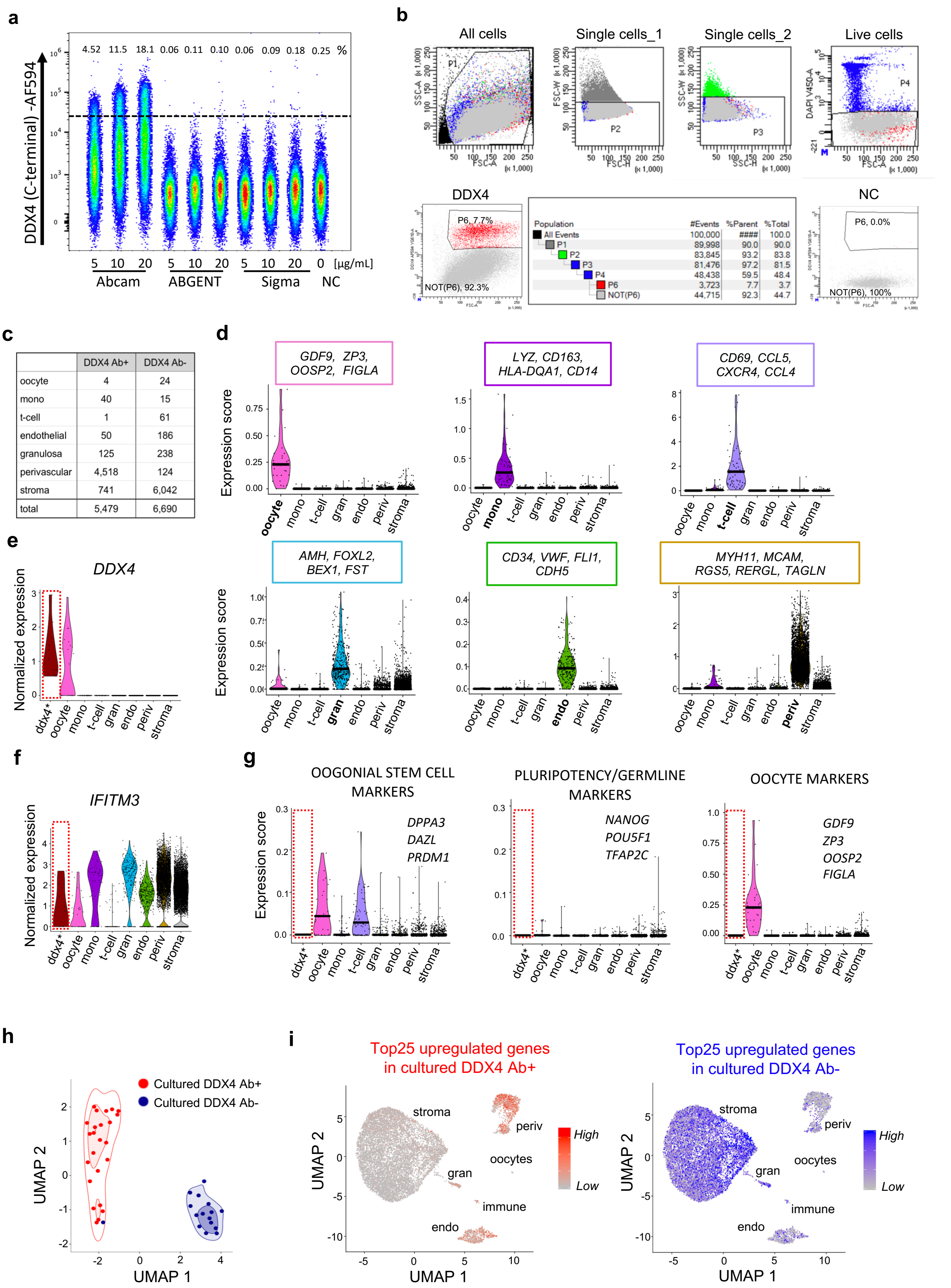

Supplementary Figure 2. Sorted ovarian DDX4 Ab+ and Ab- cortex cells. a, FACS plots of three different DDX4 C-terminus antibodies (Abcam, ABGENT, and Sigma) in three different staining concentrations (5, 10 and 20 µg/mL per sample containing max 1x10^{6} cells in 100

µL) are shown. The Abcam Ab labeled an increasing percentage of a distinct cell population (4.51% - 18.1%) with increasing concentration while ABGENT and Sigma antibodies did not. Gating of DDX4 Ab+ cells was performed on cells stained for rabbit polyclonal isotype control Ab. **b**, Representative FACS plots showing the gating strategy of dissociated ovarian tissue cells for sorting of DDX4 Ab+ cells for scRNA-seq analysis. After exclusion of debris, single cells were gated based on FSC and SSC properties. Live cells were selected based on DAPI signal. Gating of DDX4 Ab+ cells was based on FSC and distinct Ab signal. Several negative control samples were included in each sort (DAPI only, primary Ab omitted, FMO as well as rabbit polyclonal isotype control, labelled here as NC) to avoid false positive DDX4 Ab+ cells. On average, a DDX4 Ab+ cell population of 5.5 – 11.5% was observed. **c**, Contribution of cells from sorted DDX4 Ab+ and DDX4 Ab- populations to each cluster (in Fig. 3b). **d**, Color-coded violin plots showing expression scores of selected signature genes among the top highly expressed genes defining the cell type of each cluster. **e**, Somatic cells expressing *DDX4* (n=12; n=1 in Ab+ population and n=11 in Ab- population) were manually clustered to form a separate cluster (ddx4*). **f**, Violin plot showing the expression levels of *IFITM3* in all sorted ovarian cortex cells. **g**, Violin plots showing OSC (*DPPA3, DAZL, PRDM1*), pluripotency and/or germline (*NANOG, POU5F1, TFAP2C*) and oocyte markers (*GDF9, ZP3, OOSP2, FIGLA*) as expression score. None of the markers were expressed in somatic DDX4 expressing cells (except from *IFITM3).* **h**, UMAP based on single-cell transcriptomes of cultured DDX4 Ab+ and Ab- cells showing that the populations cluster separately even after extended time in cell culture in OSC culture conditions. Contour lines indicate the 2D kernel density for each cell population divided into three bins. **i**, Feature plots showing the expression score of the top 25 upregulated genes of cultured DDX4 Ab+ (scale bar depicting low expression in grey and high expression in red) and DDX4 Ab- cells (scale bar depicting low expression in grey and high expression in blue) in our freshly sequenced unsorted ovarian cells.

Horizontal bars in violin plots indicate median gene expression per cluster.

Ab, Antibody; FACS, Fluorescence Activated Cell Sorting; FMO, Fluorescence Minus One; FSC, Forward Scatter; NC, Negative Control; OSC, Oogonial Stem Cell; PCA, Principal Component Analysis; SSC, Side Scatter.

80 **Supplementary Figure 3. Validation of markers identified in transcriptomic and**
81 **proteomic analysis.**

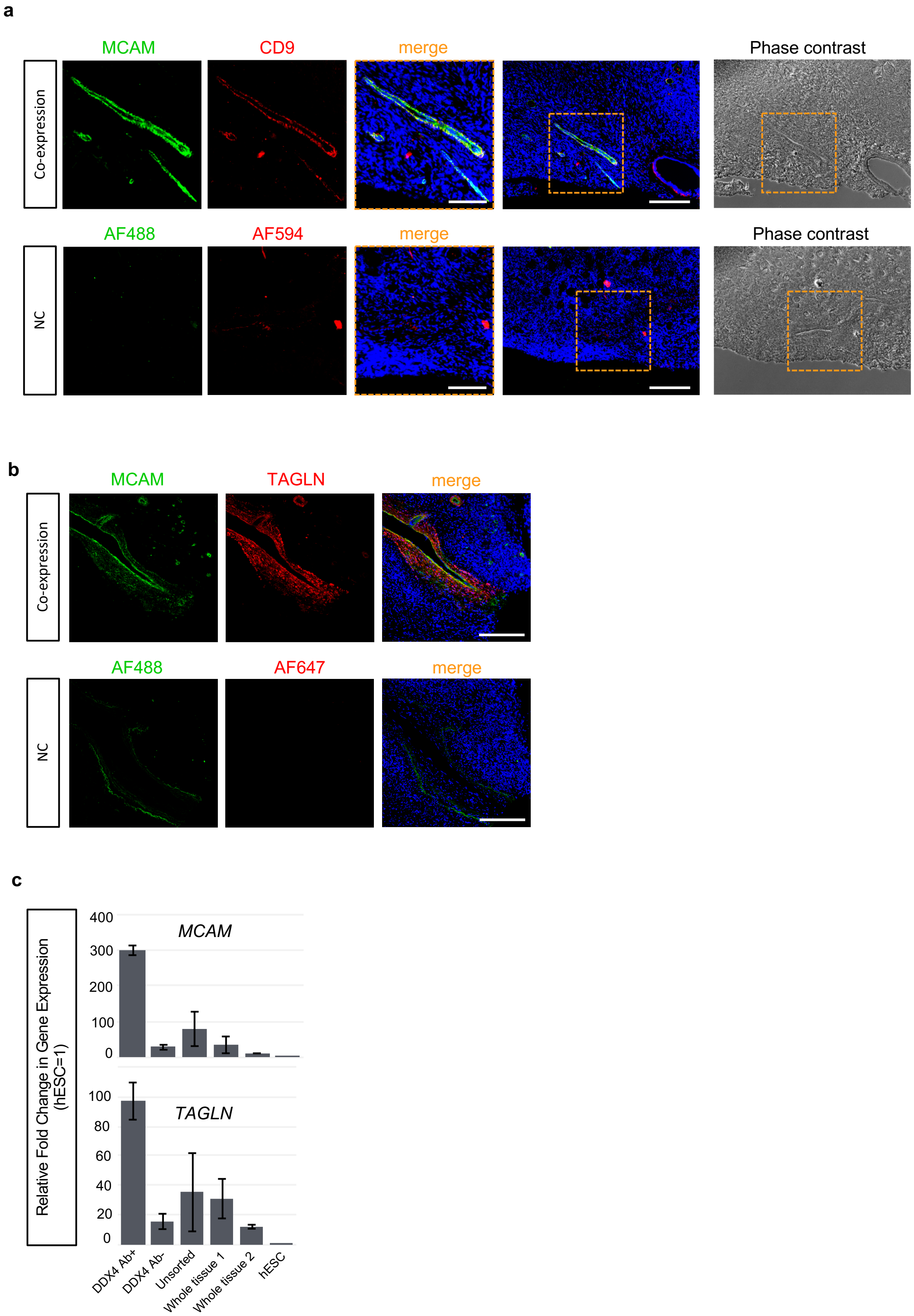

82
83

Supplementary Figure 3. Validation of markers identified in transcriptomic and proteomic analysis. **a**, Immunostaining of human ovarian cortical tissue sections from GRP donor showing the expression of MCAM (green) and CD9 (red) on a protein level. DAPI (blue) was used as nuclear counterstain. Co-expression was detected in cells of blood vessels (zoomed-out, scale bars: 200 µm). Orange rectangles demarcate the zoomed-in area (scale bars: 100 µm). Immunostaining was performed in three histological sections from three patients and a representative image is shown. **b**, Immunostaining of human ovarian cortical tissue sections from a GRP donor showing the protein expression of MCAM (green) and TAGLN (red) in cells of a blood vessel. Nuclei are counterstained using DAPI (blue). Scale bars: 200 µm. Immunostaining was performed in three histological sections from two patients. All vessels stained positive for MCAM/TAGLN and a representative image is shown. **c**, Gene expression analysis of *MCAM* and *TAGLN* in sorted ovarian tissue cells from pooled GRP donors (DDX4 Ab+/-), unsorted ovarian tissue cells (DDX4 ab+ and Ab-) from three GRP donors dissociated during independent experiments and three pieces of whole tissue from two GRP donors, respectively. Values were normalized to *GAPDH* expression and displayed relative to hESC sample. Mean ± SD is shown from three undissociated pieces of whole ovary tissue from two donors, respectively, three independently dissociated ovarian tissue samples, and three independently sorted DDX4 Ab+ and Ab- cell fractions.

As negative control (NC) in immunostaining, primary antibodies were omitted.

hESCs, human Embryonic Stem Cells.

Supplementary Figure 4. Germline-like cells in the adult human ovary.

Integration with unsorted GRP data

Integrated cluster ID	mitotic FGCs	RA resp. FGCs	meiotic FGCs	Oo-genesis	NA	endo-thelial	granulosa (w7-10)	granulosa (w10-20)	granulosa (w20-26)	GRP (5,715 cells)
mitotic FGCs	426	14	0	0	17	0	0	0	0	0
RA responsive FGCs	0	78	0	0	8	0	0	0	0	0
meiotic FGCs	2	2	90	6	27	0	0	0	0	0
oocytes/oogonia	0	0	0	25	1	0	0	0	0	3
immune	0	0	0	0	33	0	0	1	0	17
granulosa	0	0	0	0	9	0	2	2	42	1
endothelial	0	0	0	0	4	7	0	0	0	131
perivascular	12	0	0	0	14	0	2	21	2	408
stroma	5	0	3	0	67	1	33	47	120	5,155

Integration with sorted DDX4 Ab+ data

Integrated cluster ID	mitotic FGCs	RA resp. FGCs	meiotic FGCs	Oo-genesis	NA	endo-thelial	granulosa (w7-10)	granulosa (w10-20)	granulosa (w20-26)	DDX4 Ab+ (5,479 cells)
mitotic FGCs	409	1	2	0	31	0	0	25	10	1
RA responsive FGC	2	92	1	1	13	0	0	0	0	0
meiotic FGCs	0	1	89	7	26	0	0	0	0	0
oocytes/oogonia	0	0	0	23	1	0	0	0	0	2
immune	0	0	0	0	32	0	0	1	0	40
granulosa	1	0	1	0	30	1	14	3	111	124
endothelial	0	0	0	0	4	7	0	0	0	50
perivascular	32	0	0	0	29	0	0	38	24	4,495
stroma	1	0	0	0	14	0	23	4	19	767

Integration with sorted DDX4 Ab- data

Integrated cluster ID	mitotic FGCs	RA resp. FGCs	meiotic FGCs	Oo-genesis	NA	endo-thelial	granulosa (w7-10)	granulosa (w10-20)	granulosa (w20-26)	DDX4 Ab- (6,690 cells)
mitotic FGCs	437	2	2	0	24	0	1	0	0	2
RA responsive FGC	0	90	0	0	10	0	0	0	0	0
meiotic FGCs	1	2	88	8	18	0	0	0	0	0
oocytes/oogonia	0	0	0	23	1	0	0	0	0	21
immune	0	0	0	0	34	0	0	1	0	74
granulosa	0	0	2	0	30	0	3	17	79	244
endothelial	0	0	0	0	4	7	0	0	0	188
perivascular	0	0	0	0	0	0	0	0	0	108
stroma	7	0	1	0	59	1	33	53	85	6,053

Supplementary Figure 4. Germline-like cells in the adult human ovary. Our adult ovarian cortex datasets were integrated with a publicly available fetal ovary scRNA-seq dataset [1] in order to find any adult cells that would cluster with pre-meiotic germ cells. Integration was carried out sample by sample in order to avoid bias caused by disproportionate cell numbers between the datasets. Numbers of cells contributing to the integrated clusters from fetal and adult samples are shown for each comparison. Integration with unsorted C-sec sample is displayed in Fig. 5. Adult cells clustering with pre-meiotic germ cells are highlighted in red.

Supplementary Table 1. First surface marker screening of human ovarian cortex cells.

Screen 1

Surface Marker	% CD-POS	MFI
CD47	99,4	57332
CD59	99,4	99797
CD147	99,3	18778
CD55	96,3	13128
CD58	93,6	8389
CD95	83,2	8813
CD46	82,2	10896
CD29	80,4	18113
CD166	80,2	13714
CD49c	80,1	14038
CD44	79,7	8886
CD151	79,2	12902
CD63	78,5	6336
CD164	77,7	7817
CD140b	77,2	19179
CD81	77,1	7447
CD165	71,5	9929
CD73	68,1	30713
CD268	67,4	3064
CD49a	66,1	7902
B2M	65,6	10503
CD90	65,5	24181
CD321	64,6	5080
CD54	63,9	5922
CD340	63,6	3193
CD9	59,9	9134
CD26	59,8	31591
HLA-A,B,C	59,4	39782
EFR-R	59,3	3458
CD49e	57,7	9602
CD105	57,2	11868
CD62E	51	5876
CD98	50,6	2857
CD221	50,6	2538
CD200	49,5	10743
CD146	48,9	10298
HLA-DQ	46,1	4438
CD141	43,8	2941
CD61	40,3	2857
CD91	40	3508
CD142	38,1	2749
CD326	37,5	5511
CD51/61	35,1	2913
HPC	34,4	3424
HLA-A2	34,1	12284
CD338	32,5	2569
CD34	31,1	4051
CD71	30,8	2519
CD140a	30,1	2453
CD56	27,9	3500

**CD275**	26,1	2218
**CD49d**	24,3	3367
**CD107a**	23,6	2262
**CD144**	23,5	6303
**HLA-DR**	23,4	4831
**HLA-DR,DP,DQ**	21,1	2736
**CD39**	17,8	34067
**CD40**	16	2405
**CD97**	14,4	2477
**CD220**	13,6	2073
**CD49b**	13,4	2120
**CD243**	12,5	4438
**CD226**	11,9	2042
**CD227**	11,6	2588
**CD235a**	10,2	2142
**CD152**	9,59	2104
**CD183**	9,14	1996
**CD112**	8,56	1996
**CD161**	8,31	2042
**CD130**	7,97	2062
**CD107b**	7,82	2115
**CD126**	7,82	1996
**SSEA-4**	7,54	6643
**CD102**	7,25	2441
**CD77**	7,17	2690
**CD181**	6,64	2062
**CD109**	5,74	1945
**CD184**	5,66	2174
**CDw329**	5,53	1991
**CD124**	5,19	1935
**CD314**	4,92	1911
**CD271**	4,84	3334
**CD205**	4,41	2057
**CD309**	4,35	7388
**CD336**	4,32	2223
**Invariant NK**	4,25	2062
**Gamma delta TCR**	4,12	5801
**CD162**	4,03	1975
**CD11a**	4,01	2089
**CD72**	3,97	1980
**CD22**	3,91	1955
**BLTR-1**	3,72	1950
**CD66(a,c,d,e)**	3,63	3391
**CD121b**	3,6	2158
**CD13**	3,52	2837
**CD274**	3,26	2026
**CD10**	3,14	2370
**GD2**	2,91	3208
**CD4v4**	2,85	2268
**CD31**	2,77	4770
**CD27**	2,53	2115
**CD206**	2,36	2447
**CD45RA**	2,29	2037
**CD14**	2,21	2052
**CD195**	2,19	2163
**CD83**	2,18	2089
**CD197**	2,17	2201

**CD244**	2,16	6125
**CLIP**	2,15	2052
**CD231**	2,13	2324
**CD150**	2,11	2136
**CD38**	1,9	2394
**CD74**	1,89	2364
**CD36**	1,88	3508
**CD80**	1,87	2201
**CD117**	1,82	2330
**CD45RO**	1,79	2251
**CD41b**	1,74	2465
**CD134**	1,72	2207
**CD84**	1,7	2115
**CD123**	1,69	2330
**CD75**	1,65	2550
**CD53**	1,64	2613
**CD32**	1,55	2256
**CD273**	1,53	2723
**CD85**	1,49	2544
**CD177**	1,46	2273
**MIC A/B**	1,45	2163
**CD88**	1,45	2336
**CD196**	1,45	2229
**CD229**	1,39	2526
**CD62P**	1,34	2857
**CD118**	1,33	2152
**CD172b**	1,32	2229
**CD18**	1,31	2394
**CD64**	1,3	2723
**CD171**	1,3	3247
**CD57**	1,29	2507
**CD193**	1,29	2749
**CD45**	1,27	2313
**CD79b**	1,26	2347
**CD163**	1,26	2532
**CD337**	1,23	2083
**CDMR-56**	1,23	2336
**CD50**	1,23	2899
**CD137 Ligand**	1,21	2459
**CDw328**	1,2	2158
**CD15**	1,2	2417
**CD62L**	1,19	2399
**CD153**	1,17	2388
**CD278**	1,17	2913
**CD69**	1,15	2716
**NKB1**	1,11	2532
**CD178**	1,11	2697
**CD209**	1,11	2465
**CMRF-44**	1,1	2626
**CD11b**	1,09	2471
**CD45RB**	1,09	2268
**CD99**	1,09	2423
**CD158b**	1,09	2581
**CDw327**	1,08	2273
**CD279**	1,06	2411
**CD108**	1,06	2569
**SSEA-1**	1,05	2296

**CD48**	1,05	2613
**CD282**	1,03	2262
**CD5**	1,03	2899
**CD70**	1,03	2683
**CD20**	1,01	2290
**CD30**	1,01	2405
**CD35**	1,01	2405
**CD99R**	1,01	2110
**Vbeta8**	1	2229
**CD16**	1	2313
**Alpha beta TCR**	0,99	2581
**CD128**	0,99	2507
**CD305**	0,98	2223
**CDw93**	0,98	2613
**CD135**	0,97	3278
**CD66b**	0,95	2600
**CD11c**	0,94	2174
**CD43**	0,94	2756
**CD106**	0,94	2483
**fMLP-R**	0,91	2477
**CD28**	0,91	2330
**CD42a**	0,91	2483
**CD3**	0,9	2544
**CD21**	0,88	2399
**CD42b**	0,87	2489
**CD138**	0,87	3050
**CD158a**	0,87	2336
**CD33**	0,85	2330
**CD122**	0,85	2664
**CD137**	0,85	2749
**TRA-1-81**	0,84	2142
**CD1d**	0,84	2158
**CD1a**	0,82	2336
**CD8b**	0,82	2495
**CD23**	0,81	2588
**CD103**	0,81	2083
**Vbeta23**	0,8	2313
**CD66f**	0,79	2168
**CD89**	0,79	2364
**CD41a**	0,78	2207
**CD86**	0,78	2658
**C119**	0,78	2218
**CD25**	0,77	2716
**CD7**	0,76	2723
**CD154**	0,76	2501
**CD37**	0,75	2429
**CD87**	0,74	2201
**CD180**	0,74	2453
**CD255**	0,74	6875
**CD6**	0,73	2262
**CD114**	0,73	3154
**CD2**	0,72	2126
**CD1b**	0,71	2273
**CD121a**	0,71	2423
**TRA-1-60**	0,69	2519
**CD19**	0,69	2556
**CD127**	0,68	2364

**CD24**	0,67	2417
**CD100**	0,67	2613
**CD8a**	0,66	2376
**CD116**	0,66	2212
**CD335**	0,64	2313
**CD94**	0,64	2218
**CD120a**	0,62	2382
**CD4**	0,58	2353
**CD15s**	0,56	2477

Supplementary Table 1. First surface marker screening of human ovarian cortex cells. Percentage of ovarian cortex cells positive for 242 CD markers and corresponding MFI values. CD, Cluster of differentiation; MFI, Mean fluorescence intensity.

Supplementary Table 2. Second surface marker screening of human ovarian cortex cells.

Screen 2

Surface Marker	% CD-POS	MFI
CD49a	99,7	76235
CD61	99,3	467000
CD151	99,2	18996
CD56	96	16101
CD59	93	9308
CD47	87,4	9798
CD98	86,8	8263
CD141	86,5	21738
CD39	85,2	19692
CD200	83,8	17174
CD49e	83,5	15692
HLA A2	81,8	26911
CD165	81,3	13136
CD166	79	12261
CD29	74,2	27183
CD90	71,1	9798
HLA-DQ	70,5	22470
CD104	69,9	7883
CD73	69,4	5951
CD95	69,4	16953
CD26	69,1	8486
CD51/61	68,1	11002
CD63	66,5	8900
CD107a	66,5	8953
CD221	66	11559
CD81	64,5	46349
CD268	57	3003
CD49c	56,1	5310
CD336	55,6	5138
HLA-DR	53,3	5581
B2M	52,4	2979
CD55	46,7	5266
CD2	37,9	2933
HLA A,B,C	37,4	3732
CD105	34,2	2895
CD140b	31,9	2655
CD210	27,2	2979
CD147	25,4	7746
CD144	25,1	2987
CD54	23,8	3164
CD62E	23,7	3131
CD142	21,6	3139
CD340	18,4	2409
CD146	15,8	5124
HLA-DR,DP,DQ	15,7	4449
CD58	13,7	2820
CD44	13,6	41634
SSEA-4	7,94	3458
CD1b	4,81	2153

**CD120b**	2,45	5013
**CLA**	1,83	2733
**SSEA-3**	0,98	2987
**CD309**	0,93	2321
**CD267**	0,93	2941
**CD3**	0,82	2415
**CD338**	0,79	2820
**HPC**	0,76	2712
**Integrin b7**	0,7	2655
**CD321**	0,68	3018
**CD1a**	0,64	2676
**CD1d**	0,64	2513
**CD132**	0,64	2641
**CD9**	0,63	2600
**CD201**	0,62	2539
**CD294**	0,62	2655
**CD212**	0,61	2956
**CD49f**	0,33	2339
**CD46**	0	9253
**CD71**	0	7266
**gdTCR**	0	7860
**CD326**	0	5597

Supplementary Table 2. Second surface marker screening of human ovarian cortex cells. Percentage of ovarian cortex cells positive for CD markers pre-selected in the first surface marker screening (Supplementary Table 1) and corresponding MFI values. Selected CD markers determined negative in Supplementary Table 1 are included as negative controls. CD, Cluster of differentiation; MFI, Mean fluorescence intensity.

Supplementary Table 3. Third surface marker screening of human ovarian cortex cells.

Screen 3

Surface Marker	% CD-POS	MFI
CD47	99,4	55154
CD147	90,9	22055
CD55	86,6	17317
CD58	83,1	14497
CD63	72,4	10533
CD164	71,5	12249
CD46	70,1	12391
CD29	68,7	31394
CD151	68,7	23449
CD26	63,5	30331
CD95	63,4	11666
CD44	60,7	14974
CD9	59,8	18387
CD49E	58,7	19121
CD62E	57,7	18277
CD166	56	17111
HLA A,B,C	55,8	35270
CD90	54,8	33430
CD105	53,6	13997
CD49C	51,4	28230
CD140B	50,6	23812
CD54	44	9792
CD49F	41,3	11902
CD165	39,8	13875
CD200	36,7	13515
CD321	32,8	9236
CD49A	30,2	13052
CD340	30,2	6353
HLA-DQ	27,5	7212
CD221	25	6222
CD146	23,4	23739
CD201	22,7	7548
HLA-A2	19,9	29767
CD81	18,8	7467
HPC	18,3	8010
B2M	15,6	15791
CD98	14,3	6948
CD144	14	11210
CD146/MCAM	13,7	60966
CD51/61	12,1	7368
CD338	11,9	6015
HLA-DR	11,8	9365
CD39	11,6	47265
CD141	10,8	6749
CD91	10,7	6173
CD142	7,26	6488
HLA-DR,DQ,DP	7,23	8669
CD42b	5,73	6488
CD56	5,64	6141
EGF-R	5,31	5771

**CD42a**	5,31	6437
**CD107A**	5,09	5625
**CD43**	4,76	6336
**SSEA-4**	3,43	9443
**CD13**	2,42	7022
**CD77**	2,33	6505
**CD11C**	1,74	5984
**CD268**	1,61	5756
**CD4v4**	1,57	6173
**CD4**	1,17	5712
**CD326**	0,98	7135
**Gamma delta TCR**	0,66	6287

Supplementary Table 3. Third surface marker screening of human ovarian cortex cells. Percentage of ovarian cortex cells positive for CD markers pre-selected in the first surface marker screening (Supplementary Table 1) and corresponding MFI values. Selected CD markers determined negative in Supplementary Table 1 are included as negative controls. CD, Cluster of differentiation; MFI, Mean fluorescence intensity.

SUPPLEMENTARY DATA

Supplementary Data 1. Cell Ranger metrics and gene expression analysis of unsorted ovarian cortex cells.

Sheet 'Stats': Cell Ranger metrics is listed for both samples of unsorted single cells dissociated from GRP and C-sec patients' ovarian cortex, respectively. After quality control and filtering, 5,715 and 6,445 cells were kept for downstream analysis, respectively.

Sheet 'Variable genes clusters': List of markers per cluster identified using FindAllMarkers with default settings in Seurat.

Sheet 'ddx4': List of markers expressed in *DDX4* expressing somatic cells of the ovarian cortex. All markers of manually pooled somatic *DDX4* expressing cells were identified compared to all other clusters using FindMarkers with default settings in Seurat.

Supplementary Data 2. Cell Ranger metrics and gene expression analysis of sorted ovarian cortex cells.

Sheet 'Stats': Cell Ranger metrics is listed for both samples of sorted single cells dissociated from GRP ovarian cortex, DDX4 Ab+ and Ab- cells, respectively. After quality control and filtering, 5,479 and 6,690 cells were kept for downstream analysis, respectively.

Sheet 'Variable genes clusters': List of markers per cluster identified using FindAllMarkers with default settings in Seurat.

Sheet 'Variable genes Orig.ident': List of markers per sample (DDX4 Ab+ versus DDX4 Ab-) identified using FindAllMarkers with default settings in Seurat.

Sheet 'ddx4': List of markers expressed in *DDX4* expressing somatic cells of the ovarian cortex. All markers of manually pooled somatic *DDX4* expressing cells were identified compared to all other clusters using FindMarkers with default settings in Seurat.

Supplementary Data 3. Upregulated genes in cultured human ovarian DDX4 Ab+ and DDX4 Ab- cells.

Sheet 'Up Down': List of genes identified as upregulated in cultured DDX4 Ab+ and Ab- ovarian cortex cells, respectively, using featureCounts in EdgeR.

Sheet 'description': Description of all terms and abbreviations used in sheet 'Up Down'.

REFERENCES

1. Li, L., et al., *Single-Cell RNA-Seq Analysis Maps Development of Human Germline Cells and Gonadal Niche Interactions.* Cell Stem Cell, 2017. **20**(6): p. 858-873 e4.
