# Supplementary material for: Single-cell analysis of human ovarian cortex identifies distinct cell populations but no oogonial stem cells
Source: Nat Commun. 2020 Mar 2;11:1147. doi: 10.1038/s41467-020-14936-3 (PMC7052271; doi:10.1038/s41467-020-14936-3)
Supplement: Supplementary file 5 — Reporting Summary [file 41467_2020_14936_MOESM5_ESM.pdf]

nature research

Corresponding author(s): Fredrik Lanner
Pauliina Damdimopoulou

Last updated by author(s): Jan 27, 2020

Reporting Summary

Nature Research wishes to improve the reproducibility of the work that we publish. This form provides structure for consistency and transparency in reporting. For further information on Nature Research policies, see Authors & Referees and the Editorial Policy Checklist .

Statistics

For all statistical analyses, confirm that the following items are present in the figure legend, table legend, main text, or Methods section.

n/a	Confirmed	
☐	☒	The exact sample size (*n*) for each experimental group/condition, given as a discrete number and unit of measurement
☐	☒	A statement on whether measurements were taken from distinct samples or whether the same sample was measured repeatedly
☐	☒	The statistical test(s) used AND whether they are one- or two-sided<br>*Only common tests should be described solely by name; describe more complex techniques in the Methods section.*
☒	☐	A description of all covariates tested
☒	☐	A description of any assumptions or corrections, such as tests of normality and adjustment for multiple comparisons
☐	☒	A full description of the statistical parameters including central tendency (e.g. means) or other basic estimates (e.g. regression coefficient) AND variation (e.g. standard deviation) or associated estimates of uncertainty (e.g. confidence intervals)
☒	☐	For null hypothesis testing, the test statistic (e.g. *F*, *t*, *r*) with confidence intervals, effect sizes, degrees of freedom and *P* value noted<br>*Give P values as exact values whenever suitable.*
☒	☐	For Bayesian analysis, information on the choice of priors and Markov chain Monte Carlo settings
☒	☐	For hierarchical and complex designs, identification of the appropriate level for tests and full reporting of outcomes
☒	☐	Estimates of effect sizes (e.g. Cohen's *d*, Pearson's *r*), indicating how they were calculated

Our web collection on statistics for biologists contains articles on many of the points above.

Software and code

Policy information about availability of computer code

Data collection	10xGenomics: NovaSeq 6000; Smart-seq2: HiSeq 2000;<br>FACS: BD Aria Fusion and FACSDiva software v8.0.1; Flow Cytometry: CytoFLEX S and CytExpert software v2.0.0.153;<br>qPCR machine: StepOneTM real-time PCR System (Applied Biosystems);<br>Image acquisition: Nikon spinning disk confocal microscope and Nikon ultra fast widefield microscope with NIS-Elements AR software v5.11.02.
Data analysis	Cell Ranger version 2.1.1 and 3.0.1 for sample demultiplexing, barcode processing, read alignment and single cell 3’ gene counting of 10xGenomics seq data; for Smart-seq2 data, STAR_2.5.sb was used for read alignment, and featureCounts v1.5.1 for gene counting. ScRNA-seq data analysis was performed using scran v1.12.0, Seurat versions 2.3.0 and 3.0.0. All scripts were written in R v3.5.1 and can be accessed XXX.<br>FACS analysis: BD FACSDiva software v8.0.1.<br>Flow data analysis: FlowJo v10 software.<br>Image analysis: Fiji/ImageJ software v2.0.

For manuscripts utilizing custom algorithms or software that are central to the research but not yet described in published literature, software must be made available to editors/reviewers. We strongly encourage code deposition in a community repository (e.g. GitHub). See the Nature Research guidelines for submitting code & software for further information.

Data

Policy information about availability of data

All manuscripts must include a data availability statement. This statement should provide the following information, where applicable:

- Accession codes, unique identifiers, or web links for publicly available datasets
- A list of figures that have associated raw data
- A description of any restrictions on data availability

10xGenomics and Smart-seq2 scRNA-seq data have been deposited on ArrayExpress and are available under the accession codes E-MTAB-8381 and E-MTAB-8403, respectively.
Database used for scRNA-seq data of ovarian medulla (Fan et al. 2019): scRNA-seq data were accessed through Gene Expression Omnibus (GEO) using accession number GSE118127.
Database used for scRNA-seq data of human fetal gonads (Li et al. 2018): UMI count data were downloaded from http://github.com/zorrodong/germcel

Field-specific reporting

Please select the one below that is the best fit for your research. If you are not sure, read the appropriate sections before making your selection.

[x] Life sciences [ ] Behavioural & social sciences [ ] Ecological, evolutionary & environmental sciences

For a reference copy of the document with all sections, see nature.com/documents/nr-reporting-summary-flat.pdf

Life sciences study design

All studies must disclose on these points even when the disclosure is negative.

Sample size	Sample size for 10xGenomics scRNA-seq of uncultured, unsorted ovarian cells was three pooled C-sec patients and one GRP; and three pooled GRPs for uncultured, sorted ovarian cells. This sample size was determined as sufficient as resulting cell numbers (approx. 12,000 cells per data set) allowed a detection of rare cell types of 0.2 % (oocytes). In literature, oogonial stem cells are expected with a frequency of 1.7 %.<br>Sample size for Smart-seq2 scRNA-seq of sorted, cultured ovarian cells was two pooled C-sec patients.<br>Sample size for surface marker screen was five pooled GRPs for the full screen, and four and six pooled GRPs for the first and second repeat of positive markers, respectively.<br>Data validation by flow cytometry was performed on ovarian cells of three pooled GRPs.<br>Data validation by immunocytochemistry was performed on ovarian cells of three GRPs as biological replicates.<br>Data validation by immunohistochemistry was performed on ovarian tissue of two GRPs in technical triplicates, respectively.<br>Data validation by RNA-FISH was performed three times on ovarian tissue of three GRPs in technical duplicates.<br>Data validation by qPCR was performed once on ovarian tissue of at least two GRPs in technical duplicates.
Data exclusions	As part of the 10xGenomics scRNA-seq analysis, cells expressing more than 25% of mitochondrial genes (filter for poor quality cells), less than 200 (filter for poor quality cells) but more than 7000 total genes (filter for doublets) were excluded. Exclusion criteria were not pre-established but determined based on data distribution (in violin plots).
Replication	To ensure reproducibility of our data, all experiments were replicated at least twice.<br>10xGenomics scRNA-seq was performed in two independent sequencing runs using four libraries in total (two libraries in run 1 and two libraries in run 2).<br>In validation experiments, all attempts at replication were successful.<br>Independent replication was done for Fig.5b, 5c, 6a, 6b, 6d, Supplementary Fig.3a, b:<br>43 markers of the surface marker screen were repeated three times.<br>Immunocytochemistry was repeated twice.<br>Immunohistochemistry was repeated three times in technical triplicates.<br>RNA-FISH was repeated three times.<br>qPCR was performed once using three independently dissociated bulk samples and three independently sorted cell populations.
Randomization	Human samples were obtained from either C-sec patients or GRPs and used without randomization as information on tissue source was an important factor. Tissue quality was determined and only tissue rated best in quality was chosen for downstream experiments.
Blinding	FACS data were acquired by a facility. Library preparation and sequencing was performed by a facility. Investigators of scRNA-seq analyses were not blinded as. 10xGenomics scRNA-seq analysis of different samples of ovarian cortex (TGP/C-Sec; DDX4 Ab+/DDX4 Ab-) was performed using identical computational pipelines and by two researchers independently.

Reporting for specific materials, systems and methods

We require information from authors about some types of materials, experimental systems and methods used in many studies. Here, indicate whether each material, system or method listed is relevant to your study. If you are not sure if a list item applies to your research, read the appropriate section before selecting a response.

Materials & experimental systems

n/a	Involved in the study
☐	☒ Antibodies
☐	☒ Eukaryotic cell lines
☒	☐ Palaeontology
☒	☐ Animals and other organisms
☐	☒ Human research participants
☒	☐ Clinical data

Methods

n/a	Involved in the study
☒	☐ ChIP-seq
☐	☒ Flow cytometry
☒	☐ MRI-based neuroimaging

Antibodies

Antibodies used

Flow Cytometry: rabbit anti-human DDX4 (ab13840, Abcam, lot no. GR3177097-1, 10ug/mL), rabbit anti-human DDX4 (SAB1300533, Sigma-Aldrich, lot no. 101M1640, 10ug/mL), rabbit ant-human DDX4 (AP1403b, Abgent, clone RB13974, lot no. SH080130E, 10ug/mL), rabbit IgG Isotype Control (ab171870, Abcam, lot no. GR273711-2, Abcam, 10ug/mL), goat anti-human MCAM (AF932, R&D Systems, lot no. ECL0318091 and ECL031607A, 1:100), mouse anti-human CD9 (ab2215, Abcam, clone MEM-61, lot no. GR26928-18, 1:20).
Secondary antibodies: AF594 donkey anti-rabbit IgG (A21207, Thermo Fisher Scientific, lot no. 1890862, 1:250); AF488 donkey anti-goat (A11055, Thermo Fisher Scientific, lot no. 1942238, 1:250); AF594 donkey anti-mouse IgG (A21203, Thermo Fisher Scientific, lot no. 1722995, 1:250); AF647 donkey anti-rabbit IgG (A31571, Thermo Fisher Scientific, lot no. 1826679, 1:250).

Surface marker screen: BD LyoplateTM Screening Panels (560747, BD, lot no. 6342734);
Secondary antibodies: AF594 donkey anti-rabbit IgG (A21207, Thermo Fisher Scientific, lot no. XXX); AF647 anti-mouse IgG (51-9006588, BD, lot no. 607462, 1:200); AF647 anti-rat IgG (51-9006589, BD, lot no. 6074559, 1:200).

Immunostaining: rabbit anti-human DDX4 (ab13840, Abcam, lot no. GR3177097-1, 10ug/mL), goat anti-human MCAM (AF932, R&D Systems, lot no. 1827674, 1:200), mouse anti-human CD9 (312102, BioLegend, clone HI9a, lot no. B201819, 1:200), mouse anti-human RGS5 (MA5-25584, Thermo Fisher Scientific, clone OTI1C1, lot no. UE2765581, 1:50); rabbit anti-human CD31 (M0823, Dako, clone JC70A, lot no. 20065916, 1:200); mouse anti-human TAGLN (MAB78861, R&D systems, clone 859112, lot no. CHXP021903A, 1:100).
Secondary antibodies: AF488 donkey anti-goat (A11055, Thermo Fisher Scientific, lot no. 1942238, 1:250); AF594 donkey anti-mouse IgG (A21203, Thermo Fisher Scientific, lot no. 1722995, 1:250); AF647 donkey anti-rabbit IgG (A31571, Thermo Fisher Scientific, lot no. 1826679, 1:250). All secondary antibodies were used in a dilution of 1:1000 for immunocytochemistry and of 1:200 for immunohistochemistry.

Validation

All antibodies used are commercially available and were produced to detect the respective human antigen. Specificity of antibodies used was assured in our experiments by detecting the reported localization of its antigen (blood vessels and oocyte structures can be detected in phase contrast) as well as by using negative controls.
BD Lyoplate Screening Panels contain antibodies validated for flow cytometry by the manufacturer.
MCAM antibody: was tested for immunoreaction with human embryonic stem cells by cytochemistry and with cells in human melanoma tissue by histochemistry by the manufacturer. This antibody was cited by six more publications.
Abcam CD9 antibody: tested for immunohistochemistry and flow cytometry by manufacturer. This antibody was used in 17 citations.
Biolegend CD9 antibody: validated for immunocytochemistry by manufacturer. This antibody was cited in five more publications.
RGS5 antibody: validated for immunohistochemistry by manufacturer.
CD31 antibody: More than 400 citations used this antibody, widely used for endothelial cell detection (i.e. Orlova et al. 2014, Nat Prot).
TAGLN antibody: tested for immunocytochemistry in human breast epithelial cell line by manufacturer. Not validated for immunohistochemistry. In our hands, the antibody clearly labeled cells surrounding vessels indicative of perivascular cell detection.
Abcam DDX4 antibody: tested for flow cytometry, immunohisto- and -cytochemistry; this product has been cited in more than 300 publications and was used in the original article on oogonial stem cells (White et al. 2012).
Sigma-Aldrich DDX4 antibody: antibody was produced to detect C-terminal domain of DDX4. Was not validated for flow cytometry by the manufacturer but tested for immunohistochemistry.
ABGENT DDX4 antibody: antibody was produced to detect C-terminal domain of DDX4. Was not validated for flow cytometry by the manufacturer but tested for immunohistochemistry.

Eukaryotic cell lines

Policy information about cell lines

Cell line source(s)

HS980, human embryonic stem cell line, derived and cultured as stated in Rodin et al. Nature Communications, 2014 (5). Embryos for stem cell derivation were collected with written informed consent from both parents after approval by the Swedish Ethical Review Authority (Dnr. 2011/745:31/3).

Authentication	No authentication procedure.
Mycoplasma contamination	HS980 cell line was routinely tested negative for mycoplasma contamination.
Commonly misidentified lines (See ICLAC register)	No commonly misidentified cell lines were used.

Human research participants

Policy information about studies involving human research participants

Population characteristics	Ovarian cortical tissue from 16 gender reassignment patients (age range 20 – 38 yr) and five Cesarean section patients (age range 28 – 37 yr) was used. Prior to gender reassignment surgery, all gender reassignment patients underwent androgen treatment for 14 – 89 months.
Recruitment	Independent clinicians informed the patients about the study, and cortical tissue was biopsied from Cesarean section patients and whole ovaries collected from gender reassignment patients after written informed consent.
Ethics oversight	Use of ovarian tissue in research was approved by Stockholm Region Ethical Review Board (Dnr. 2010/549-31/2, Dnr. 2015/798-31/2).

Note that full information on the approval of the study protocol must also be provided in the manuscript.

Flow Cytometry

Plots

Confirm that:

- [x] The axis labels state the marker and fluorochrome used (e.g. CD4-FITC).
- [x] The axis scales are clearly visible. Include numbers along axes only for bottom left plot of group (a 'group' is an analysis of identical markers).
- [x] All plots are contour plots with outliers or pseudocolor plots.
- [x] A numerical value for number of cells or percentage (with statistics) is provided.

Methodology

Sample preparation	Human ovarian tissue was thawed, cut into smaller pieces using scalpels and enzymatically digested in medium containing Collagease IA and Liberase in a shaking 37°C water bath for max. 50 min. Filtered single cell suspension was blocked on 4°C for 20 min, followed by incubation with primary antibodies on 4°C for 20 min. After washing, cells were incubated with secondary antibodies on 4°C for 20 min. After final washing, cells were resuspended in buffer containing DAPI.
Instrument	FACS software: BD Aria Fusion; Flow Cytometry software: CytoFLEX S
Software	BD FACSDiva software; FLowJo v10 software
Cell population abundance	Cells were sorted based on DDX4 signal. On average, a DDX4 Ab+ cell population of 5.5 – 11.5% was observed. Varying cell population abundance was observed when different CD markers were used (see Figure 4a).
Gating strategy	Cells were gated based on FSC-A/SSC-A for P1, then FSC-W/FSC-H (P2) and SSC-W/SSC-H (P3) for singlets. DAPI negative, live cell population (P4) was used to further analyze/sort DDX4 positive (P6) and negative (NOT (P6)) cells. Exemplified gating strategy in Supplementary Figure 2b.

- [x] Tick this box to confirm that a figure exemplifying the gating strategy is provided in the Supplementary Information.
