# Supplementary material for: Single-cell analysis of human ovarian cortex identifies distinct cell populations but no oogonial stem cells
Source: Nat Commun. 2020 Mar 2;11:1147. doi: 10.1038/s41467-020-14936-3 (PMC7052271; doi:10.1038/s41467-020-14936-3)
Supplement: Supplementary file 6 — Description of Additional Supplementary Files [file 41467_2020_14936_MOESM6_ESM.pdf]

Title: Supplementary Data 1
Description: Cell Ranger metrics and gene expression analysis of unsorted ovarian cortex cells.

Title: Supplementary Data 2
Description: Cell Ranger metrics and gene expression analysis of sorted ovarian cortex cells.

Title: Supplementary Data 3
Description: Upregulated genes in cultured human ovarian DDX4 Ab+ and DDX4 Ab- cells.
